# Integrating lifestyle and clinical data in prostate cancer: expert assessment of a questionnaire

**DOI:** 10.1007/s00345-026-06494-y

**Published:** 2026-05-22

**Authors:** Catarina Leitão, Luís Monteiro, Margarida Fardilha, Fátima Roque, Maria Teresa Herdeiro

**Affiliations:** 1 Department of Medical Sciences, Institute of Biomedicine (iBiMED), University of Aveiro, Campus Universitário de Santiago, Aveiro, 3810-193 Portugal; 2 CINTESIS – Centre for Health Technology and Services Research, Faculdade de Medicina, Universidade do Porto, Porto, Portugal; 3 Department of Medical Sciences, University of Aveiro, Campus Universitário de Santiago, Aveiro, 3810-193 Portugal; 4 USF Esgueira +, ULS Região Aveiro, Aveiro, 3800-322 Portugal; 5 BRIDGES - Biotechnology Research, Innovation and Design for Health Products, Polytechnic University of Guarda, Avenida Dr. Francisco Sá Carneiro, n. 50, Guarda, 6300- 559 Portugal

**Keywords:** Prostate cancer, Questionnaire development, Expert assessment, Lifestyle factors, Dietary habits, Clinical data

## Abstract

**Purpose:**

Prostate cancer (PCa) remains a significant health burden as one of the most common cancers among men worldwide. Given the growing evidence of lifestyle’s impact on PCa progression, this study evaluates multidisciplinary experts’ feedback on a novel questionnaire assessing lifestyle factors potentially influencing PCa progression.

**Methods:**

Developed through systematic literature reviews and insights from focus groups with clinical practice professionals, namely interns and specialists in urology and family medicine (FM), the questionnaire covers four domains: sociodemographic data, dietary habits, lifestyle factors, and clinical data.

**Results:**

The questionnaire was evaluated by 25 professionals from the fields of public health, epidemiology, urology, and family medicine (FM), as well as by two linguists and two clinical psychologists. The questionnaire was first evaluated using a 5-point Likert scale for adequacy, accuracy, completeness, format, interest, usefulness, and confidence. Subsequently, each domain was also assessed individually for adequacy, accuracy, and completeness, using the same Likert scale, with qualitative feedback refining the questionnaire. Overall, this questionnaire received positive evaluations, with a mean score of 4.47 ± 0.72 and a median score of 4 or higher for each assessment. All domains achieved median scores of 5 for adequacy and accuracy, though completeness scored slightly lower in the lifestyle domain. Qualitative feedback highlighted the questionnaire’s relevance and clarity, while suggesting minor refinements.

**Conclusion:**

These findings underscore the potential of this structured data collection protocol for exploring lifestyle-PCa associations, supporting future case-control studies, and advancing targeted prevention and intervention strategies for PCa risk management.

**Supplementary Information:**

The online version contains supplementary material available at 10.1007/s00345-026-06494-y.

## Introduction

Prostate cancer (PCa) is the fourth most frequently diagnosed malignancy and the eight leading cause of cancer-related mortality worldwide, with over one million cases reported in 2022 [1]. Its global burden is particularly significant in Europe and Asia, accounting for over 30% of diagnoses and deaths, respectively [1, 2]. While established risk factors such as age, race, family history, and genetic predisposition are non-modifiable, emerging evidence highlights the growing impact of modifiable lifestyle factors on PCa development and progression. Dietary habits, physical activity, alcohol consumption, and smoking are increasingly recognized as contributors to chronic inflammation [3, 4] and oxidative stress, mechanisms implicated in cancer development [3, 5].

Despite the growing interest in lifestyle and cancer prevention, studies investigating the impact of lifestyle on PCa risk have yielded inconsistent results [3, 6, 7], partly due to methodological heterogeneity and limitations in data collection instruments. There are numerous questionnaires commonly used with patients with PCa that serve as fundamental tools for assessing various aspects related to the disease. Although these studies do not provide specific examples of standardized questionnaires, such as the International Prostate Symptom Score (IPSS) or the Expanded Prostate Cancer Index Composite (EPIC), they still offer valuable information regarding the main domains typically assessed by these instruments. Many existing tools, however, are limited in scope, focusing primarily on symptoms, treatment side effects, and quality of life (QoL), as exemplified by the questionnaires mentioned above. Additionally, few instruments are specifically tailored to the unique context of PCa. For instance, the IPSS is widely used but primarily designed for patients with benign prostatic hyperplasia (BPH) [8, 9], as it focuses mainly on urinary symptoms. In contrast, the EPIC, developed for PCa patients, comprises 50 questions covering five clinical domains: urinary incontinence, obstructive urinary symptoms, intestinal symptoms, sexual symptoms, and hormonal symptoms, each scored separately [10, 11]. Additionally, there are questionnaires and risk calculators specifically designed to evaluate PCa risk factors. These tools incorporate a range of variables including age, family history, ethnicity, urinary symptoms and results of clinical tests such as prostate-specific antigen (PSA) levels and digital rectal examination (DRE) findings [12, 13]. However, regarding modifiable risk factors, existing tools are limited in scope, often assessing only individual lifestyle domains (e.g., diet or physical activity) [14–16].

To address this gap, we developed a questionnaire covering four key domains: sociodemographic data, dietary habits, lifestyle, and clinical factors (see Online Resource 1). The primary aim of this study is to evaluate the feedback from health professionals, linguists, and clinical psychologists obtained during an expert-based face and content validation of the questionnaire. This evaluation seeks to identify areas for improvement, ensuring the tool’s clarity, relevance, and alignment with current knowledge on PCa risk factors. Ultimately, this assessment may support future research, by providing a comprehensive tool that may help streamline data collection regarding modifiable risk factors and contribute to preventive and intervention strategies.

## Materials and methods

### Ethical considerations

This study consisted of an online expert review of a research questionnaire, by appraising the wording, structure, and content of each item and by providing methodological feedback (e.g., adequacy, accuracy, completeness, usefulness, and format). Experts were not asked to complete the questionnaire as respondents. Therefore, no health or clinical data, personal experiences, or sensitive opinions were collected from participants, beyond professional judgement regarding the instrument’s content. Beyond professional judgement about the instrument’s content, only a set of background variables was collected to describe the panel and explore subgroup patterns (age, gender, professional area, and years of professional experience). No direct identifiers (e.g., name, email address, IP address) were collected, and data were analyzed and reported only in an aggregated form.

Given the minimal-risk nature of this expert-review activity and the absence of any intervention, the study protocol and data processing plan were reviewed by the University of Aveiro’s Data Protection Officer (DPO) of the institution, ensuring that the study complies with the General Data Protection Regulation (GDPR).

Participation in the study was contingent upon informed consent embedded within the form. Before accessing the evaluation form, participants reviewed the study’s objectives and consent terms and were informed of their right to withdraw from completing the evaluation form at any time without consequences.

### Questionnaire development

The questionnaire (provided in Online Resource 1) was designed to identify potential modifiable risk factors for PCa, structured across four domains: sociodemographic data, dietary habits, lifestyle and clinical factors.

Its development followed a multi-step process aimed at ensuring both clinical relevance and contextual appropriateness, drawing upon two prior studies: a narrative literature review [3] and a focus group (FG) study with clinical practice professionals [17]. While the literature review provided a broad overview of the associations between lifestyle, inflammation, and PCa, the FG study contributed more directly to item generation. Conducted with specialists and interns in urology and general practice/family medicine (GP/FM), it explored beliefs about key PCa risk factors, barriers to lifestyle change among patients, and existing gaps in professional training. These insights helped shape the questionnaire’s content by highlighting clinically relevant themes and real-world challenges in PCa prevention.

### Study design and participants

An expert-based evaluation was conducted to evaluate the questionnaire’s clarity, comprehensiveness, and relevance. Healthcare professionals from diverse fields, including GP/FM, pharmacology, public health, epidemiology and clinical psychology, and linguistics experts were recruited for their expertise in assessing the questionnaire.

### Data collection

Data collection was conducted online to ensure accessibility for participants. The questionnaire, under review, developed for application in the male population, was made available in PDF format. To obtain expert feedback on this instrument, an evaluation form (provided in Online Resource 2) was created using FormsUA, in anonymous mode, which utilizes LimeSurvey software for online form implementation.

Both the questionnaire (attached as a PDF file) and the evaluation form link were distributed via institutional email lists from health institutions and academic departments, rather than through individualized contact by the research team. Participants accessed the evaluation form through a secure link provided in the invitation email and provided responded using FormsUA.

### Face and content validity assessment

To ensure its clarity and relevance, face and content validity were assessed through expert review, through a combined quantitative and qualitative approach. Face validity was established by evaluating clarity, comprehensibility, and relevance, while content validity was reinforced through expert feedback to ensure domain comprehensiveness. This expert-driven approach, supported by systematic and narrative reviews, as well as FG insights, minimized ambiguity and enhanced the questionnaire’s robustness.

For the assessment process, participants were recruited and asked to complete an online evaluation form. This form included: (1) collection of participants’ sociodemographic data (gender, age, education level, professional area, and years of experience); (2) five groups of closed questions aiming to quantitatively evaluate the general and domain-specific contents and elements of the questionnaire, using a 5-point Likert scale (1: Strongly Disagree to 5: Strongly Agree); and (3) five open-ended questions aimed at collecting qualitative feedback for refining ambiguous items and improving content coverage. The structure of this evaluation form was based on a previously published approach [18]. Experts provided feedback on each domain—sociodemographic data (7 items), dietary habits (20 items), lifestyle data (17 items), and clinical data (40 items)—ensuring the comprehensiveness of the instrument (Table 1).

**Table 1:** Domains of the questionnaire evaluated and its content

1. Sociodemographic data	Questions related to age, gender, educational background, and professional area
2. Dietary habits	Questions designed to capture detailed information on participants’ dietary patterns, including frequency of consumption of specific food groups
3. Lifestyle data	Questions on physical activity levels, smoking habits, and alcohol consumption
4. Clinical data	Questions related to clinical history and other relevant health indicators

By combining quantitative and qualitative evaluation methods, this assessment approach ensured that the questionnaire was well-structured, scientifically robust, and capable of capturing relevant lifestyle and clinical factors associated with PCa.

### Statistical analysis

All statistical analyses were performed using GraphPad Prism 8.0. Descriptive statistics analyses were performed to illustrate demographic characteristics and to quantitatively evaluate the questionnaire’s content, both overall and within specific domains. Results were reported as mean ± standard deviation (SD), median, the 25th and 75th percentiles, mode and range. Additionally, to gain a better insight about the participant’s opinions, comments, and suggestions overall, as well as to clarify possible comprehensible problems, the qualitative feedback provided by the experts was evaluated.

## Results

### Participants’ characteristics

The questionnaire was assessed by 29 professionals, including 25 experts from the fields of public health, epidemiology, urology, and general practice/family medicine (GP/FM), as well as by two linguists and two clinical psychologists. Participants’ characteristics are summarized in Table 2 and Online Resource 3 – Figure S1.

**Table 2:** Participants’ characteristics

Characteristics	*n*	Percentage (%)
Total participants	29	100.0
Men	11	37.9
Women	18	62.1
Age		
26–35	10	34.5
36–45	8	27.6
46–55	5	17.2
56–67	6	20.1
Educational level		
Bachelor’s degree	6	20.7
Postgraduate degree	1	3.4
Master’s degree	14	48.3
Doctoral degree	8	27.6
Professional area		
Health sciences	25	86.2
Family medicine (FM)	18	72.0
Pharmacology/epidemiology/public health	6	24.0
Urology	1	4.0
Social sciences	4	13.8
Clinical psychology	2	50.0
Linguistics	2	50.0
Professional experience		
0–9 years	10	34.5
10–19 years	6	20.7
20–29 years	7	24.1
30–39 years	3	10.3
40–49 years	3	10.3

Most participants were women (62.1%), and the median age of all participants was 42 years old, with the most frequent group being 26–35 years old (34.5%). Regarding educational background, most participants held a Master’s degree (48.3%) followed by those with a Doctoral degree (27.6%). Moreover, 20.7% of participants had a Bachelor’s degree, while fewer than 5% held a Postgraduate Degree. Professional experience ranged widely (mean of 18.24 ± 12.88 years), with 34.5% of participants reporting less than 10 years, 24.1% between 20 and 29 years of experience, and 10.3% more than 30 years of experience.

### Questionnaire evaluation

#### Descriptive analysis

The overall assessment of the face and content validity of the questionnaire parameters, conducted by the experts, is displayed in Table 3 and Online Resource 3 – Figures S2 and S3. All 29 participants who completed the evaluation form rated the questionnaire highly, with a mean score of 4.47 (± 0.72) on a 5-point Likert scale. This outcome was derived from the responses concerning adequacy, accuracy, completeness, format, utility, interest, and confidence provided by the study participants. Most participants found the questionnaire to be adequate, accurate, and in line with the literature, since 69% of participants rated adequacy and accuracy with a median score of 5.00, while 72.4% rated format with the same median score.

**Table 3:** Experts’ evaluation of the overall characteristics’ contents and domain-specific contents

General evaluation	Parameters	Median (PCT25, PCT75)	Mode	Range (Min-Max)
	Adequacy	5.00 (4.00, 5.00)	5	2–5
	Accuracy	5.00 (4.00, 5.00)	5	3–5
	Completeness	4.00 (4.00, 5.00)	4	2–5
	Format	5.00 (4.00, 5.00)	5	3–5
	Usefulness	5.00 (4.00, 5.00)	5	3–5
	Interest	4.00 (4.00, 5.00)	5	3–5
	Confidence	5.00 (4.00, 5.00)	5	1–5

Domain	Parameters	Median (PCT25, PCT75)	Mode	Range (Min-Max)
Sociodemographic data	Adequacy	5.00 (5.00, 5.00)	5	3–5
	Accuracy	5.00 (4.50, 5.00)	5	3–5
	Completeness	5.00 (5.00, 5.00)	5	3–5
Dietary habits	Adequacy	5.00 (4.50, 5.00)	5	3–5
	Accuracy	5.00 (5.00, 5.00)	5	3–5
	Completeness	5.00 (4.00, 5.00)	5	3–5
Lifestyle	Adequacy	5.00 (4.00, 5.00)	5	2–5
	Accuracy	5.00 (4.00, 5.00)	5	2–5
	Completeness	4.00 (4.00, 5.00)	5	2–5
Clinical data	Adequacy	5.00 (4.00, 5.00)	5	3–5
	Accuracy	5.00 (4.50, 5.00)	5	3–5
	Completeness	5.00 (4.00, 5.00)	5	3–5

Regarding the evaluation of separate domains that compose the questionnaire, all four domains were individually graded with a median score of 4 or above regarding their adequacy, accuracy and completeness, which reflects some homogeneity of the answers among experts (Table 3). Adequacy, accuracy, and completeness all received strong scores (median score of 5.0), with the completeness of the lifestyle domain slightly lower (median score of 4.0).

Sociodemographic and dietary habits domains were particularly well-rated, with over 75% of participants rating them as adequate and accurate, while lifestyle and clinical data reached 68%. Completeness scores varied, with dietary habits and clinical data domains rated slightly lower (58.6%).

#### Qualitative feedback

To complement the quantitative data, participants provided qualitative feedback, regarding strengths and areas for improvement (Table 4).

Experts praised the questionnaire’s relevance, clarity, user-friendly format, and well-structured information, as one participant noted, “Accessible language and editable PDF questionnaire. Very comprehensive” (Table 4 and Online Resource 3 – Table S1). Some experts suggested additional questions regarding physical activity duration, alternative tobacco products, and dietary antioxidants (Table 5). While questions on vasectomy procedures and illicit substance use were incorporated, since this information is readily available in clinical records and aligns with the study’s objectives, the suggestion to evaluate the frequency of antioxidant consumption was not implemented, as the dietary domain focuses on broader food categories rather than specific items. Technical refinements focused mainly on improving clarity and usability, including clarifying terminology, adjusting response options, reordering items in the dietary habits domain, and removing duplicate choices. The final version of the questionnaire is provided in Online Resource 4. Many respondents also acknowledged the potential of the questionnaire to aid in establishing correlations between lifestyle factors and PCa, although some expressed caution regarding causality due to limitations such as sample size and confounding factors. Regarding the question “Considering the goal of the study mentioned above, do you think this multifaceted questionnaire could be effective in establishing a possible association between lifestyle and the onset and/or development of PCa?”, experts generally agreed that its comprehensive approach to various behavioral factors could provide valuable insights into potential associations. Nevertheless, some experts emphasized the need for further studies to validate these associations and strengthen the evidence base.

**Table 4:** Qualitative feedback from participants regarding the questionnaire

Question	Theme	Answer example	Mentions (*n*)
What did you like the most about the questionnaire?	Format and simplicity	*“Accessible language and editable PDF questionnaire. Very comprehensive”*	21
	Content relevance	*“I think the questionnaire addresses the main topics related to lifestyle*,* allowing for a complete characterization”*	11
	Practical usefulness	*“Practical to answer*,* highly relevant topic in clinical practice and subject to intervention”*	4
What did you like the least about the questionnaire?	Format and length	“It’s a bit long.”	6
	Clarity and information gaps	*“Regarding the characterization of physical exercise*,* it would also be important to assess the duration of activity*,* not just frequency”*	4
	Barriers to response	*“Some questions are quite technical*,* requiring guidance from someone administering the questionnaire”*	6
	Question relevance	*“Difficult to understand the relevance of some questions*,* particularly some analytical values that won’t be considered routine tests - e.g.*,* ionogram”*	7
Do you feel that this questionnaire could bring benefits in terms of PCa prevention? If yes, in what way? If not, what are the reasons?	Identification of risk factors and lifestyle relations	*“Yes*,* because it presents hypotheses of various risk factors for the pathology that are often neglected (Nutrition*,* exercise*,* and lifestyles).”*	11
	Prevention and awareness benefits	*“I think it is a questionnaire that will allow observing some associations between lifestyle and PCa*,* which may contribute to raising public awareness of the importance of lifestyle measures for their health.”*	9
	Scientific validation and relevance of results	*“Regardless of the outcome of this study*,* assessing the relationship between lifestyle habits and PCa will always contribute to clarifying the etiologies of PCa. Regarding its prevention*,* only a comprehensive intervention at the social and economic level can bring benefits.”*	11

**Table 5:** Observations/suggestions for the questionnaire improvement

Question’s clarity
(i) “A suggestion to add an option “Other situation” for individuals who are not registered as unemployed but are currently at home without employment.”
(ii) “The phrasing of the profession-related question could be revised to “If employed, state your profession” to make it more specific.”
(iii) “The question “How many years have you been in your current profession?” may be challenging for those who have been in the profession for only a few months.”
(iv) “The term “frequent” in the question about alcohol consumption needs a clearer definition (e.g., does it mean daily or weekly consumption? ).”
(v) “For participants who no longer smoke but have smoked in the past, it was suggested to add a section asking “How many years?” they smoked.”

Additional questions
“A question on whether the participant has undergone a vasectomy, as some studies suggest a link between the procedure and PCa.”
“Expanding tobacco-related questions to include the use of pipes, cigars, or cigarillos.”
“Adding questions regarding the use of illicit substances or other dependencies.”
“Evaluating the frequency of antioxidant consumption, particularly foods like tomatoes (lycopene), pomegranates, broccoli, and green tea, all of which may have protective effects against PCa.”

## Discussion

This study assessed the face and content validity of a comprehensive questionnaire integrating sociodemographic, dietary, lifestyle, and clinical domains. Unlike previous tools focusing on isolated lifestyle factors, such as diet [15, 16], physical activity [14], alcohol consumption [19, 20], or smoking habits [21, 22], this questionnaire offers a holistic assessment, capturing the interplay of multiple influences on PCa risk and progression [23]. By addressing this gap, it enables a broader and more interconnected evaluation of lifestyle influences on PCa risk. However, for effective application in research and clinical practice, questionnaires should be relevant, clear, and practical [24, 25].

Expert assessment confirmed the questionnaire’s face and content validity, with overall positive feedback across domains. The structured assessment process, which combined expert review, evidence from systematic [26] and narrative reviews [3], and focus group insights [17], ensured that all four domains were comprehensive and clinically relevant. All domains received median scores of 4 or higher for adequacy, accuracy, and completeness, reinforcing the questionnaire’s robustness. Sociodemographic and dietary domains were particularly well-rated, with over 75% of experts considering them adequate and accurate. The lifestyle domain was also positively evaluated, though the completeness scores showed slightly more variability, possibly due to expert suggestions for additional refinements.

The qualitative feedback provided by experts offered valuable insights into specific areas for improvement. Common suggestions included refining the wording of some questions, particularly those related to physical activity and alcohol consumption, where terms were sometimes perceived as ambiguous. Additionally, experts proposed expanding response options to capture more nuanced variations in dietary habits and smoking behaviors. These suggestions also included more detailed questions on physical activity duration, diverse tobacco product use (e.g., cigars, pipes), and dietary antioxidant intake. While questions on vasectomy and illicit substance use were incorporated, given their availability in clinical records and relevance to the study, the recommendation to assess antioxidant intake frequency was not implemented, as the dietary domain focuses on broader food categories rather than specific items. Despite these suggestions, experts consistently emphasized the clarity, relevance, and applicability of the questionnaire, praising its structured format and ease of use, as revealed by the qualitative feedback.

When compared with existing tools, the added value of this questionnaire becomes evident. Established PCa risk calculators, such as the European Randomized Study of Screening for Prostate Cancer (ERSPC) Risk Calculator [27], CanRisk-Prostate Tool [28], and the Prostate Cancer Prevention Trial Risk Calculator (PCPTRC) Version 2.0 [29], primarily focus on clinical and genetic factors, offering valuable but limited perspectives. Additionally, some questionnaires in this field only evaluate patients’ quality of life after treatment [30], such as the Prostate Cancer Quality of Life scale (Pc-QoL) [31], the European Organization for Research and Treatment of Cancer, Quality of Life Questionnaire for prostate cancer patients (QLQ-PR25) [32, 33] or the University of California—Los Angeles Prostate Cancer Index (UCLA-PCI) [34]. However, lifestyle-based questionnaires, such as the Lifestyle Assessment Tool for Cancer [35] or others [36] provide only partial insights by incorporating factors like diet, physical activity, and smoking, but often omit clinical or genetic data and are not always tailored to PCa risk assessment, potentially limiting their applicability in this field. In contrast, this structured questionnaire simultaneously evaluates multiple domains, enabling a more comprehensive and personalized risk assessment. Its design allows healthcare professionals to identify lifestyle patterns that may influence PCa development, creating opportunities for prevention strategies. Moreover, the questionnaire can be instrumental in future research, enabling the collection of standardized lifestyle data in large-scale epidemiological studies.

This study assessed face and content validity through an expert-based mixed-methods approach, combining quantitative Likert-scale ratings (e.g., adequacy, accuracy, completeness, usefulness, and format) with qualitative open-ended feedback. Content validity was not quantified using the Content Validity Index (CVI), and additional measurement properties were not employed, which may limit conclusions about reproducibility and measurement performance under field conditions. Importantly, this questionnaire was not designed as a psychometric scale intended to measure latent behavioral or psychological constructs or to generate summed domain scores [25, 37]. Instead, it was developed as a structured data-collection tool to capture sociodemographic characteristics, objective lifestyle exposures and clinical/history variables that are intended to be analyzed primarily as individual items [25, 37]. This differs from symptom and health-related quality of life instruments commonly used in prostate conditions (e.g., IPSS and EPIC), which are psychometric scales explicitly designed for scoring and construct-level validation [8–11]. Accordingly, some scale-development procedures (e.g., factor analysis and internal consistency) do not apply to most items in the present tool [25, 37]. In addition, many lifestyle questions are intentionally framed to capture participants’ usual/habitual patterns without imposing an explicit reference period; therefore, test–retest agreement is difficult to interpret because differences between administrations could reflect true behavioral fluctuation or differences in how respondents operationalize “usual,” rather than measurement error [37]. For record-based clinical variables, data are obtained by extracting the most recent available value for each requested parameter within the previous 12 months; thus, data quality depends primarily on documentation and standardized extraction rules rather than on psychometric properties [38, 39]. Similar expert-driven methods have been effectively applied in previous questionnaire-development studies, particularly when the primary aim is to ensure content adequacy, clarity, and feasibility prior to field implementation. Since this questionnaire was developed primarily for use by research teams or healthcare professionals, its design prioritizes clarity and feasibility in expert-administered settings rather than direct patient self-completion.

Despite its strengths, additional limitations must be acknowledged. The questionnaire relies on self-reported data for diet and lifestyle, which may introduce recall and social desirability bias. The limited representation of urologists, reflecting the lower participation from this specialty in our dissemination channels, may have influenced domain-specific feedback, particularly for the clinical data domain. PCa specialists may prioritize different variables, levels of technical detail, and feasibility considerations compared to other professionals. However, the clinical domain was informed by the literature and by a prior focus group study [17], and was structured to include a broad range of variables at this stage. It incorporates both PCa-specific clinical variables and routinely collected clinical parameters, including standard hematology and biochemistry panels, reflecting a more holistic approach to patient assessment, aligned with clinical practice and European Association of Urology (EAU) guidelines [40]. As lifestyle factors may influence these biomarkers, their inclusion supports a more integrated analysis of PCa progression. Nevertheless, the relevance and availability of specific tests may vary across settings, highlighting a trade-off between comprehensive coverage and practical feasibility when applying the instrument.

Future research could integrate objective lifestyle measures, such as wearable activity trackers or dietary intake biomarkers, to complement self-reported responses and strengthen data reliability. By refining this questionnaire, it can serve as a valuable resource for lifestyle-PCa research, ensuring standardized and consistent data collection in epidemiological and clinical studies. Future studies should compare its effectiveness with existing risk assessment tools, test its applicability across diverse populations, and refine specific domains based on further expert and user feedback [41, 42]. Overall, this study provides a comprehensive questionnaire for assessing lifestyle factors associated with PCa, supporting targeted prevention strategies and advancing research on modifiable risk factors. Its integration of multiple domains allows for a comprehensive evaluation of risk factors, supporting targeted prevention efforts.

## Supplementary Information

Below is the link to the electronic supplementary material.

Supplementary Material 1

Supplementary Material 2

Supplementary Material 3

Supplementary Material 4

## Data Availability

The data underlying this article is available in the article and in its online supplementary material.
