# Supplementary material for: Integrating lifestyle and clinical data in prostate cancer: expert assessment of a questionnaire
Source: World J Urol. 2026 May 22;44(1):376. doi: 10.1007/s00345-026-06494-y (PMC13197278; doi:10.1007/s00345-026-06494-y)
Supplement: Supplementary file 1 — Supplementary Material 1 [file 345_2026_6494_MOESM1_ESM.pdf]

Integrating Lifestyle and Clinical Data in Prostate Cancer: Assessment of a Questionnaire through a Pilot Study

Catarina Leitão [1, *], Luís Monteiro [2,3,4], Margarida Fardilha [1], Fátima Roque [5] and Maria Teresa Herdeiro [1]

[1] Department of Medical Sciences, Institute of Biomedicine (iBiMED), University of Aveiro, Campus Universitário de Santiago, 3810-193 Aveiro, Portugal; mfardilha@ua.pt (M.F.); teresaherdeiro@ua.pt (M.T.H.)

[2] CINTESIS – Centre for Health Technology and Services Research, Faculdade de Medicina, Universidade do Porto. Porto, Portugal; monteiroluis@ua.pt (L.M.)

[3] Department of Medical Sciences, University of Aveiro, Campus Universitário de Santiago, 3810-193 Aveiro, Portugal

[4] USF Esgueira +, ULS Região Aveiro, 3800-322 Aveiro, Portugal

[5] Biotechnology Research, Innovation and Design for Health Products (BRIDGES), Research on Epidemiology and Population Health Laboratory, Polytechnic of Guarda, Avenida Dr. Francisco Sá Carneiro, 6300-559 Guarda, Portugal

*Correspondence: catarinaileitao@ua.pt

Questionnaire Evaluation Form for the Male Population

Prostate cancer (PCa) is the most common cancer and the 3rd most lethal cancer in men, accounting for 20% of estimated new cancers and 10.5% of cancer-related deaths. Despite the difficulty in determining the etiology of PCa, there are several well-established risk factors, such as patient age, chronic inflammation associated with advanced age, black race, family history, and genetic alterations.

In recent years, various studies have reported that environment and lifestyle may be directly associated with chronic inflammation and could lead to the onset and/or progression of PCa. However, since this correlation is not yet well established, the study to be conducted is a part of a work plan whose main objective is to assess the impact of lifestyle on this neoplasm, with a greater focus on nutritional status and inflammation.

To carry out this stage, a qualitative study in the form of focus groups was previously conducted to understand the perceptions and experiences of specialists and interns in General and Family Medicine and Urology. Based on the results obtained, a questionnaire was developed to be administered to the male population. Thus, a pilot study was designed to validate the questionnaire.

The form for the present study is divided into three parts. The first part, consisting of four brief questions, will collect sociodemographic data such as age, profession, years of experience, and level of education. The second part contains four groups of closed-ended questions, and the third part consists of four open-ended questions, both aimed at evaluating the elements of the questionnaire.

We thank you in advance for your collaboration. If you need any further clarification about the study, please do not hesitate to contact the Responsible Researcher at the email: catarinaileitao@ua.pt

Thank you very much,

Catarina Leitão

This work was funded by a doctoral research grant with reference UI/BD/151352/2021

This survey is anonymous.

The recording of survey responses does not contain any information about your identity, except if a survey question specifically asks for identification and you provide it. If you used a code to access this survey, that code will not be stored with your responses. The code is managed in a separate database and is only used by the program to record that you completed the survey. There is no way to link the codes of those invited to participate in the survey with the responses given.

Informed Consent

This study is part of the doctoral thesis project "Impact of Nutritional Status and Inflammation on Prostate Cancer" with reference UI/BD/151352/2021 funded by FCT. Its main objective is to validate a questionnaire to be administered to the male population regarding their lifestyle.

The study complies with the General Data Protection Regulation (GDPR), ensuring the security and confidentiality of all data provided by the participants, as well as guaranteeing that the responses provided will never be associated with their identity. All collected data will be encrypted and electronically stored, and only the responsible researcher will have access to it. The person responsible for handling and collecting your data is Catarina Isabel Moreira Leitão, a student of the Doctoral Program in Biomedicine at the University of Aveiro. It is also assured that all data will be completely destroyed after the dissemination of results, expected by December 2024. The results will be disclosed exclusively in a scientific context (in presentations or publications) and will be treated in an aggregated form, never individually.

Consent is voluntary, and it is possible to withdraw from the study at any time. You can contact the responsible researcher for the project, Catarina Leitão (iBiMED-UA), by email at catarinaileitao@ua.pt if you wish to clarify any doubts, and you can also contact the Data Protection Officer of the University of Aveiro at epd@ua.pt. If necessary, you may also file a complaint with the National Data Protection Commission at cnpd.pt.

*** I declare that I have read and understood the information contained in this document and that I have been duly informed and clarified about the objectives and conditions of participation in this study, and that as such:**

□ I declare under GDPR and LGDP that I GIVE my consent to participate in the study.

□ I declare under GDPR and LGDP that I DO NOT GIVE my consent to participate in the study.

Sociodemographic Data

*Age:

*Gender:

- Female
- Male

*Educational Qualifications
Select all that apply

□ Bachelor's Degree

□ Postgraduate

□ Master's Degree

□ Doctoral Degree

*Professional Area:
Select all that apply

□ General and Family Medicine

□ Pharmacology

□ Epidemiology

□ Public Health

□ Clinical Psychology

□ Linguistics

□ Outro:

* How many years have you been practicing your profession?

General Evaluation Measures of the Questionnaire: Impact of Lifestyle on Prostate Cancer

Please rate each of the following parameters using the scale provided:
☆ Strongly Disagree ☆☆ Disagree ☆☆☆ Neutral ☆☆☆☆ Agree ☆☆☆☆☆ Strongly Agree

* **Adequacy:** I consider the contents and information provided by the questionnaire to be adequate.

* **Accuracy**: I consider the contents and information provided by the questionnaire to be accurate and in accordance with the literature.

* **Completeness:** I consider the contents and information provided by the questionnaire to be complete.

* **Format**: The presentation of this content in editable .pdf format is adequate.

* **Usefulness:** I consider the information collected in this questionnaire to be useful and that it can contribute to better understanding the impact of lifestyle on the progression of this neoplasm.

* **Interest**: I would recommend the use of this questionnaire to a colleague.

* **Confidence:** I am confident that the questions formulated in the questionnaire can contribute to establishing a possible correlation between lifestyle and prostate cancer.

Evaluation of the Constituent Sections of the Questionnaire: Impact of Lifestyle on Prostate Cancer

Please rate each of the following parameters using the scale provided:
☆ Strongly Disagree ☆☆ Disagree ☆☆☆ Neutral ☆☆☆☆ Agree ☆☆☆☆☆ Strongly Agree

Section 1 - Sociodemographic Data

***Adequacy:** I consider the contents of this module to be adequate.

***Accuracy:** I consider the contents of this module to be accurate and in accordance with the literature.

***Completeness:** I consider the contents of this module to be complete.

Section 2: Dietary Habits Data

***Adequacy:** I consider the contents of this module to be adequate.

***Accuracy:** I consider the contents of this module to be accurate and in accordance with the literature.

***Completeness:** I consider the contents of this module to be complete.

Section 3: Lifestyle Data

***Adequacy:** I consider the contents of this module to be adequate.

***Accuracy:** I consider the contents of this module to be accurate and in accordance with the literature.

***Completeness:** I consider the contents of this module to be complete.

Section 4: Clinical Data

***Adequacy:** I consider the contents of this module to be adequate.

***Accuracy:** I consider the contents of this module to be accurate and in accordance with the literature.

***Completeness:** I consider the contents of this module to be complete.

Open-ended Questions

Questionnaire "Impact of Lifestyle on Prostate Cancer":

***What did you like most about the questionnaire?**

***What did you like least about the questionnaire?**

***Do you think this questionnaire could bring benefits in terms of prostate cancer prevention?**

If yes, in what way?

If no, what are the reasons?

Observations/comments regarding the questionnaire "Impact of Lifestyle on Prostate Cancer":

* In Portugal, Prostate cancer (PCa) accounts for 20% of estimated new cancers and 10.5% of cancer-related deaths. The implementation of prostate-specific antigen (PSA) detection allowed PCa to be detected and treated at an early stage of development. However, false positives sometimes occur, leading to unnecessary interventions that can potentially cause side effects such as sexual impotence or urinary incontinence. Therefore, it is necessary to consider new biomarkers that may be found through the analysis of other risk factors that are not yet well associated. Thus, this questionnaire aims to establish an association between these factors and PCa to identify new biomarkers to replace and/or complement the use of PSA.

Considering the goal of the study mentioned above, do you think this multifaceted questionnaire could be effective in establishing a possible association between lifestyle and the onset and/or development of PCa?
