# Supplementary material for: Integrating lifestyle and clinical data in prostate cancer: expert assessment of a questionnaire
Source: World J Urol. 2026 May 22;44(1):376. doi: 10.1007/s00345-026-06494-y (PMC13197278; doi:10.1007/s00345-026-06494-y)
Supplement: Supplementary file 2 — Supplementary Material 2 [file 345_2026_6494_MOESM2_ESM.docx]

**Integrating Lifestyle and Clinical Data in Prostate Cancer: Assessment of a Questionnaire through a Pilot Study**

Catarina Leitão ^1, *^, Luís Monteiro ^2,3,4^, Margarida Fardilha ^1^, Fátima Roque ^5^ and Maria Teresa Herdeiro ^1^

^1^ Department of Medical Sciences, Institute of Biomedicine (iBiMED), University of Aveiro, Campus Universitário de Santiago, 3810-193 Aveiro, Portugal; [mfardilha@ua.pt](mailto:mfardilha@ua.pt) (M.F.); [teresaherdeiro@ua.pt](mailto:teresaherdeiro@ua.pt) (M.T.H.)

^2^ CINTESIS – Centre for Health Technology and Services Research, Faculdade de Medicina, Universidade do Porto. Porto, Portugal; [monteiroluis@ua.pt](mailto:monteiroluis@ua.pt) (L.M.)

^3^ Department of Medical Sciences, University of Aveiro, Campus Universitário de Santiago, 3810-193 Aveiro, Portugal

^4^ USF Esgueira +, ULS Região Aveiro, 3800-322 Aveiro, Portugal

^5^ Biotechnology Research, Innovation and Design for Health Products (BRIDGES), Research on Epidemiology and Population Health Laboratory, Polytechnic of Guarda, Avenida Dr. Francisco Sá Carneiro, 6300-559 Guarda, Portugal

*Correspondence: [catarinaileitao@ua.pt](mailto:catarinaileitao@ua.pt)


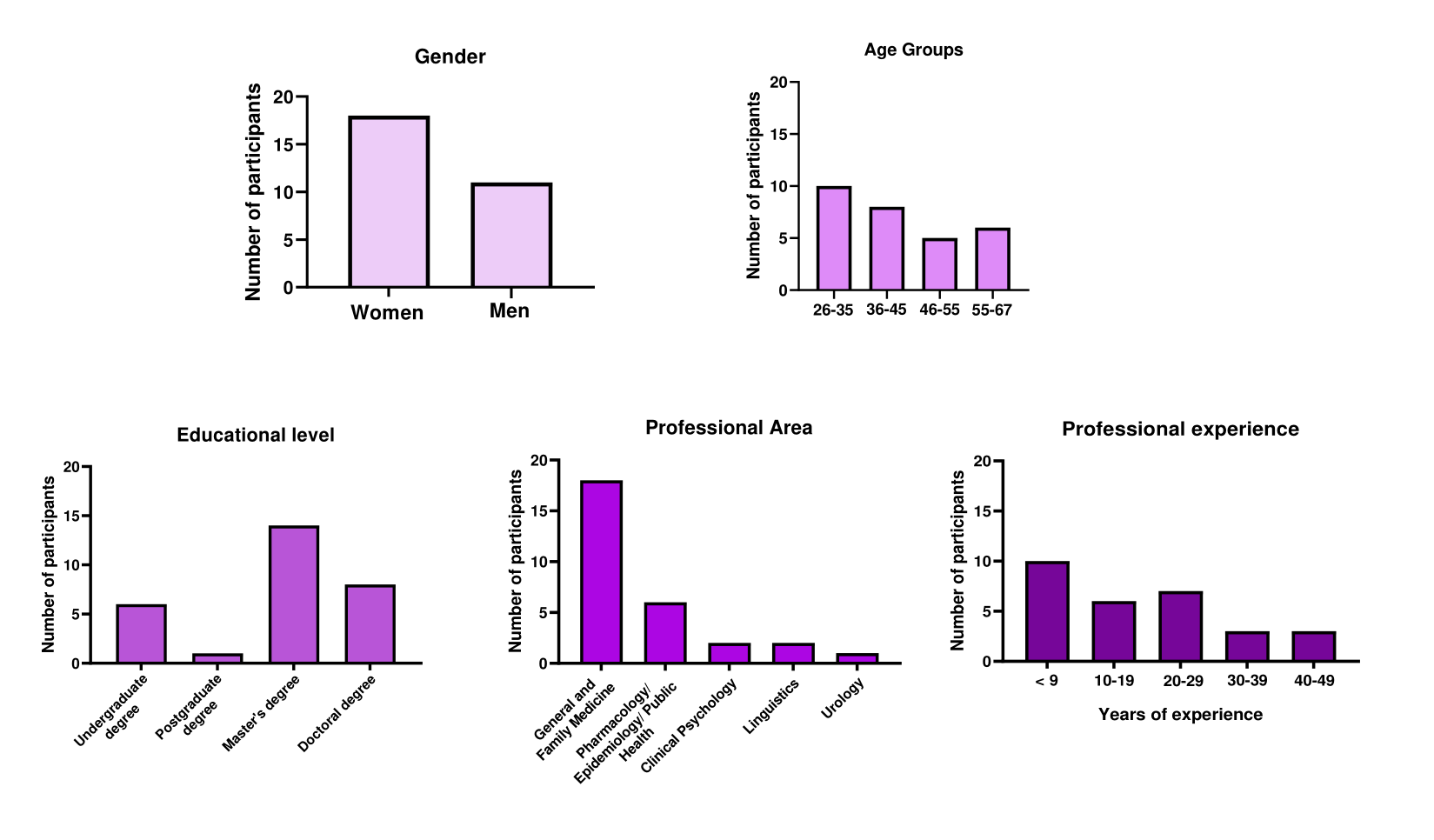


**Figure S1** - Frequencies of the distribution of demographic data of participants evaluating the questionnaire (n=29).


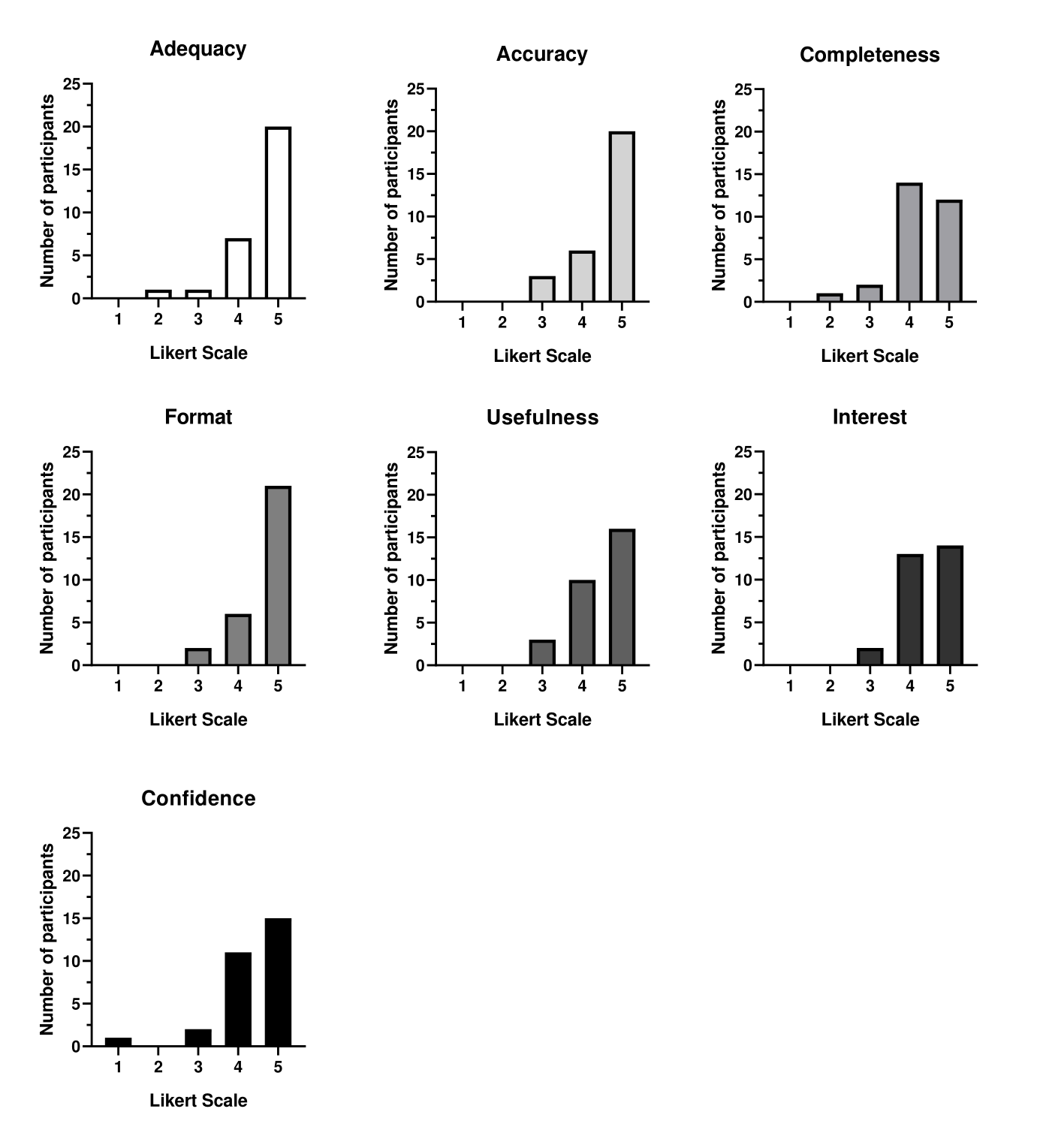


**Figure S2** - Frequencies of responses on the general evaluation of the questionnaire (n= 29).


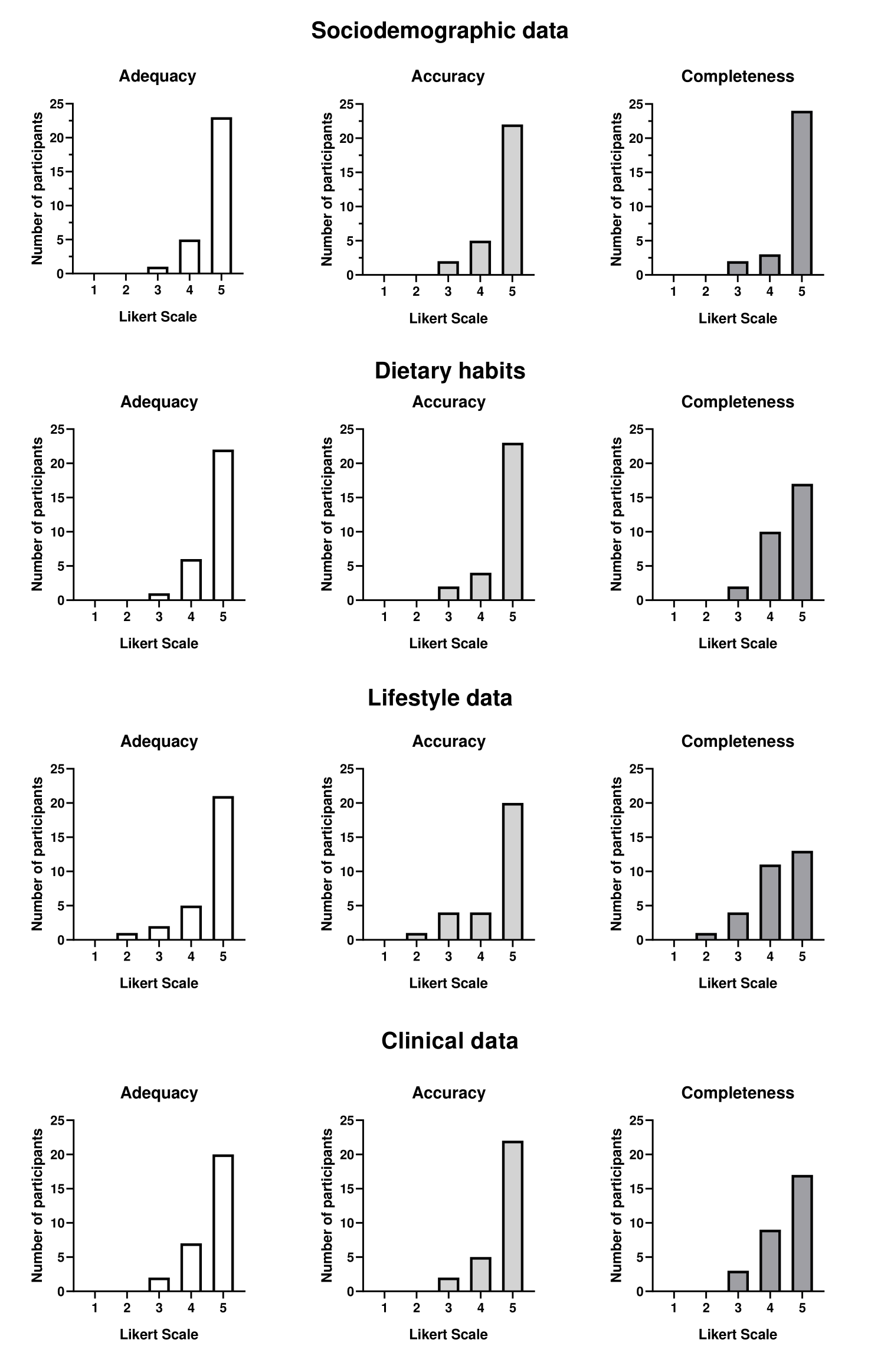


**Figure S3** - Frequencies of the rates given to each parameter of each module of the questionnaire (n=29).

**Table S1** - Qualitative feedback from participants.

| **What did you like the most about the questionnaire?** | **Theme** |
| --- | --- |
| ***“The sequence of the questions.”*** | **Format and Simplicity** |
| ***"The fact that it is an online questionnaire (with large/visible letters) and is an editable PDF."*** | **Format and Simplicity** |
| ***“Accessible language and editable PDF questionnaire. Very comprehensive. Covers all relevant aspects and risk factors in prostate cancer."*** | **Format and Simplicity; Content Relevance** |
| ***"I think the questionnaire addresses the main topics related to lifestyle, allowing for complete characterization. The last part regarding clinical data seems quite complete."*** | **Content Relevance** |
| ***“Carefully presented and easy to fill out."*** | **Format and Simplicity** |
| ***"Very targeted questions with direct answers."*** | **Format and Simplicity** |
| ***"Simplicity."*** | **Format and Simplicity** |
| ***"The fact that the questions are very direct and easy to interpret."*** | **Format and Simplicity** |
| ***"Simple and easy to complete questionnaire."*** | **Format and Simplicity** |
| ***"Objectivity and the study itself of causality."*** | **Practical Usefulness** |
| ***"Alcohol consumption, food."*** | **Content Relevance** |
| ***"Practical to answer, highly relevant topic in clinical practice and subject to intervention."*** | **Format and Simplicity; Practical Usefulness** |
| ***"I liked the clear and organized structure of the questionnaire, which facilitates understanding and filling it out."*** | **Format and Simplicity** |
| ***"Speed and clarity."*** | **Format and Simplicity** |
| ***"I think the part about clinical data and tobacco characterization."*** | **Content Relevance** |
| ***"Theme."*** | **Content Relevance** |
| ***"The .pdf format is very useful in terms of ease of completion. It also seems to me that the questionnaire is quite complete, although I suggest some changes."*** | **Format and Simplicity; Content Relevance** |
| ***"Organization of information and easy to understand."*** | **Format and Simplicity; Practical Usefulness** |
| ***"Intuitive, easy to fill out overall."*** | **Format and Simplicity** |
| ***"Objectivity and ease of filling out."*** | **Format and Simplicity** |
| ***"Simple questions that are easy for patients to answer."*** | **Format and Simplicity** |
| ***"Easy to answer and practical."*** | **Format and Simplicity** |
| ***"Simplicity of the questions, especially regarding habits and lifestyles."*** | **Format and Simplicity; Content Relevance** |
| ***"Practical."*** | **Practical Usefulness** |
| ***"Specificity of questions."*** | **Content Relevance** |
| ***"The .pdf format."*** | **Format and Simplicity** |
| ***"Everything, the format facilitates filling out the questionnaire; it seems quite complete to me."*** | **Format and Simplicity; Content Relevance** |
| ***"It is very specific for the various questions."*** | **Content Relevance** |
| ***"Easy to fill out, very specific and direct questions*** | **Format and Simplicity; Content Relevance** |
| **What did you like the least about the questionnaire?** | **Theme** |
| *“The length, the high number of questions.”* | Format and Length |
| *“I think that given the diversity of questions, this would make more sense in a semi-structured interview format than in a questionnaire (although I understand the logic, objective, and relevance of it). Let's not forget that a good portion of these patients may have difficulties filling it out online (low digital literacy) due to age.”* | Format and Length; Barriers to response |
| *“In section "3. Lifestyle Data," the part about indicating how many glasses per week you consume of each drink.”* | Question relevance |
| *“I can't say it's something I liked less, but I have a suggestion: In section "3. Lifestyle Data," wouldn’t it make sense to characterize whether there is consumption of other drugs? Since they ask about alcohol and tobacco?”* | Clarity and Information gaps |
| *“Nothing”* | - |
| *“The length of the questionnaire”* | Format and Length |
| *“It should be avoided to consider tobacco consumption as a lifestyle question; in reality, it is an addiction. It would be better to ask "Do you have any sexually transmitted diseases?" also regarding the past: "Have you had any sexually transmitted diseases in the past?" And to add a question: "Have you had any prostate disease or infection in the past?"* | Clarity and Information gaps |
| *“The questions regarding types of food”* | Question relevance |
| *“The three questions from each module are similar.* | Question relevance |
| *“Nothing to report”* |  |
| *“The determination of BMI by survey will not be necessary. Better to ask for height and weight. I believe some risk factors are missing, such as sexual practices.”* | Question relevance |
| *“Regarding the analyses, it may not be the quickest to answer! This factor may interfere with adherence.”* | Barriers to response |
| *“Not applicable”* | - |
| *“Not applicable. I liked everything.”* | - |
| *“Regarding the characterization of physical exercise, it will also be important to characterize the duration of activity and not just the frequency. Regarding the question "Have you ever consumed alcoholic beverages frequently," it would be important to detail what is considered "frequently." Most of the Portuguese population will respond no, because they consider that they do not drink frequently; the Portuguese undervalue what they drink.”* | Clarity and Information gaps |
| *“Long”* | Format and Length |
| *“It is a bit lengthy.”* | Format and Length |
| *“Everything seems very appropriate to me.”* | - |
| *“Some parameters are very discriminative and have various temporal options, which makes filling out difficult and time-consuming. For example, the number of times for different types of food is difficult to understand the relevance of some questions, namely some analytical values that will not be considered routine analyses - e.g., ionogram.”* | Barriers to response; Question relevance |
| *“The request for the description of the ultrasound.”* | Question relevance |
| *“Asking a patient specifically if they have or have had any sexually transmitted disease may lead to untruthful or evasive answers. Regarding auxiliary diagnostic tests, some items seem to me to be of little relevance, and other items, on the other hand, are absent.”* | Barriers to response; Question relevance |
| *“Nothing”* | - |
| *“Filling method - Filling out a form would make it more practical for the user. Some questions are quite technical, requiring guidance from the person administering the questionnaire.”* | Barriers to response |
| *“I would say that some specific information is missing for dietary and lifestyle data.”* | Clarity and Information gaps |
| *“It will be complicated for users to answer certain questions without proper guidance.”* | Barriers to response |
| *“There is nothing I disliked.”* | - |
| *“There is nothing I didn't like.”* | - |
| *“It is very long.”* | Format and Length |
| *“Not applicable.”* | - |
| **Do you feel that this questionnaire could bring benefits in terms of prostate cancer prevention? If yes, in what way? If not, what are the reasons?** | **Theme** |
| *“Yes, it allows identifying risk factors associated with prostate cancer.”* | Scientific Validation and Relevance of Results |
| *“The questions seem relevant to me; however, since I do not work in the field of oncological pathology, I do not have as much sensitivity to assess. I would suggest that the experts to be consulted from the various areas listed be people who specifically work with this population.”* | Scientific Validation and Relevance of Results |
| *“Yes. Despite scientific and clinical advancements, the risk factors associated with this pathology are still not fully understood, and current diagnostic methods are not very specific or sensitive. This questionnaire could provide new data on the existence or not of a relationship between environmental, genetic, and/or behavioral factors and prostate cancer. More and better information will help men make more informed decisions about their lifestyle and the use of diagnostic methods.”* | Identification of Risk Factors and Lifestyle Relations; Prevention and Awareness Benefits |
| *“I think this questionnaire will allow observing some associations between lifestyle and prostate cancer, which may contribute to raising awareness in the population about the importance of lifestyle measures for their health.”* | Prevention and Awareness Benefits |
| *“Yes. By exploring various factors that may condition a higher risk of prostate cancer, with which there is still no proven relationship.”* | Identification of Risk Factors and Lifestyle Relations |
| *“I believe so, as it allows for greater knowledge of pathologies and habits associated with this condition.”* | Identification of Risk Factors and Lifestyle Relations |
| *“It depends on the results and conclusions.”* | Scientific Validation and Relevance of Results |
| *“Yes, addressing the problem helps alert the population to change habits and the need for screenings.”* | Prevention and Awareness Benefits |
| *“Yes, because it presents hypotheses of various risk factors for the pathology that are often neglected (nutrition, exercise, and lifestyle).”* | Identification of Risk Factors and Lifestyle Relations |
| *“Yes. It can reflect a cause-and-effect relationship, paving the way for the implementation of guidelines for prostate cancer prevention.”* | Prevention and Awareness Benefits |
| *“Yes! If it is possible to perceive a potential causal relationship, we can intervene at different prevention stages.”* | Prevention and Awareness Benefits |
| *“Yes. This questionnaire has the potential to identify significant correlations between lifestyle and the risk of developing prostate cancer, which can lead to more effective preventive interventions.”* | Identification of Risk Factors and Lifestyle Relations |
| *“Health literacy and advancing knowledge.”* | Scientific Validation and Relevance of Results |
| *“Of course, but the questions need to be more detailed to facilitate establishing correlations and obtaining statistically significant results that can be treated quantitatively.”* | Scientific Validation and Relevance of Results |
| *“If they manage to obtain significant results or at least some trends, I think it can only bring benefits in terms of prevention.”* | Scientific Validation and Relevance of Results |
| *“Yes, since screening questions about lifestyle allow feedback on whether the person is at risk or not.”* | Prevention and Awareness Benefits |
| *“Yes - understanding behavior profiles that can help establish causal/risk relationships and for which preventive measures can be established in the future.”* | Identification of Risk Factors and Lifestyle Relations; Prevention and Awareness Benefits |
| *“I believe it will bring benefits as it may potentially allow for adopting a better lifestyle.”* | Identification of Risk Factors and Lifestyle Relations |
| *“Regardless of the result of this study, evaluating the relationship between lifestyle habits and prostate cancer will always contribute to clarifying the etiologies of prostate cancer. Regarding prevention, only a global intervention at the social and economic level can bring benefits, as normally all powers, including political ones, are subordinate to economic interests.”* | Scientific Validation and Relevance of Results |
| *“Yes, if they verify the existence of significant differences in any factor.”* | Scientific Validation and Relevance of Results |
| *“Confirming the suspected relationship between cancer and lifestyle habits makes sense to investigate for the promotion of habits and lifestyles that prevent its onset.”* | Prevention and Awareness Benefits |
| *“Of course, if significant differences can be proven, it could be very useful.”* | Scientific Validation and Relevance of Results |
| *“Lifestyle influences other pathologies, so if a clear relationship is demonstrated, it will be beneficial since we can advise users accordingly.”* | Identification of Risk Factors and Lifestyle Relations |
| *“I don't know if it will be possible to create associations.”* | Scientific Validation and Relevance of Results |
| *“Yes, if it can prove the relationship of any of the elements beyond age, race, and family history, there could be good advancement in this area.”* | Scientific Validation and Relevance of Results |
| *“I believe so, as it can allow for the identification of risk factors that are not currently identified.”* | Identification of Risk Factors and Lifestyle Relations |
| *“Yes, even if a direct relationship cannot be shown, there may be inferences about what could impact this pathology more, and consequently, it could create some alert among the population.”* | Identification of Risk Factors and Lifestyle Relations; Prevention and Awareness Benefits |
