# Supplementary material for: Integrating lifestyle and clinical data in prostate cancer: expert assessment of a questionnaire
Source: World J Urol. 2026 May 22;44(1):376. doi: 10.1007/s00345-026-06494-y (PMC13197278; doi:10.1007/s00345-026-06494-y)
Supplement: Supplementary file 4 — Supplementary Material 4 [file 345_2026_6494_MOESM4_ESM.pdf]

Integrating Lifestyle and Clinical Data in Prostate Cancer: Assessment of a Questionnaire through a Pilot Study

Catarina Leitão [1,*], Luís Monteiro [2,3,4], Margarida Fardilha [1], Fátima Roque [5] and Maria Teresa Herdeiro [1]

[1] Department of Medical Sciences, Institute of Biomedicine (iBiMED), University of Aveiro, Campus Universitário de Santiago, 3810-193 Aveiro, Portugal; mfardilha@ua.pt (M.F.); teresaherdeiro@ua.pt (M.T.H.)

[2] CINTESIS – Centre for Health Technology and Services Research, Faculdade de Medicina, Universidade do Porto. Porto, Portugal; monteiroluis@ua.pt (L.M.)

[3] Department of Medical Sciences, University of Aveiro, Campus Universitário de Santiago, 3810-193 Aveiro, Portugal

[4] USF Esgueira +, ULS Região Aveiro, 3800-322 Aveiro, Portugal

[5] Biotechnology Research, Innovation and Design for Health Products (BRIDGES), Research on Epidemiology and Population Health Laboratory, Polytechnic of Guarda, Avenida Dr. Francisco Sá Carneiro, 6300-559 Guarda, Portugal

*Correspondence: catarinaileitao@ua.pt

Questionnaire: Impact of Lifestyle on Prostate Cancer

Questionnaire No:

Date of questionnaire completion:

1. Sociodemographic Data

Age: years **Municipality of Residence:**

Which of the following options best defines the group you consider yourself to belong to?

Select one of the options

What is the highest level of education you have completed? Select one of the options

Employment Status: Select one of the options **Profession:**

For how many years have you been in your current profession?

2. Dietary Habits Data

How often do you consume the following foods?

White Meats (chicken, turkey, duck, rabbit): Select one of the options

Red Meats (beef, pork, goat, lamb): Select one of the options

Processed Meat Products (ham, prosciutto, bacon): Select one of the options

Plant-based Products (soy, tofu, seitan, tempeh): Select one of the options

Cured Meats: Select one of the options

Fresh fish: Select one of the options

Canned fish: Select one of the options

Eggs: Select one of the options

Dairy products: Select one of the options

Fresh fruit: Select one of the options

Cannedd fruit: Select one of the options

Vegetables: Select one of the options

Legumes: Select one of the options

Snacks (sweet or salty): Select one of the options

Fast-food/Pre-prepared Meals: Select one of the optio[illegible]

Natural juices: Select one of the options

Soft Drinks: Select one of the options

Tea: Select one of the options

Coffee: Select one of the options

Water: Select one of the options

3. Lifestyle Data

1. Do you engage in any physical activity? ◯ Yes ◯ No

If yes, please select all suitable options:

Individual sport: ☐ Please specify which:

Group sport: ☐ Please specify which:

Cardiovascular/ aerobic training (walks, runs, yoga, pilates) ☐ Please specify which:

Strength training ☐

If yes, please indicate the frequency:: Select one of the options

2. Have you ever frequently consumed alcohol beverages? Yes ◯ No ◯

Do you currently consume any of these drinks?

Beer:	Yes ◯	No ◯	If yes, how many glasses per week?
White wine:	Yes ◯	No ◯	If yes, how many glasses per week?
Red wine:	Yes ◯	No ◯	If yes, how many glasses per week?
Cognac	Yes ◯	No ◯	If yes, how many glasses per week?
Appetizers:	Yes ◯	No ◯	If yes, how many glasses per week?
Spirits:	Yes ◯	No ◯	If yes, how many glasses per week?
Other drinks:	Yes ◯	No ◯	If yes, how many glasses per week?

3. Do you currently smoke? Yes ◯ No ◯ Have you ever smoked? Yes ◯ No ◯

If yes, please specify:

- Type of tobacco smoked: Select one of the options
- Year of start of consumption:
- Number of cigarettes/day
- Number of years of consumption:

4. Clinical data (using medical records)

Weight (kg): BMI: Abdominal perimeter (cm):

Systolic blood pressure [SBP (mmHg)]: Diastolic blood pressure [DBP (mmHg)]:

Do you have any sexually transmitted diseases? Yes ◯ No ◯

If yes, please specify

Medication prescribed in the last 12 months (according to the international non-proprietary name (INN):

Do you have or have you had a first-degree relative with prostate cancer? Yes ◯ No ◯

Family history regarding other pathologies:

Profile of the last generic and routine exams performed

Hematology

Erythrocytes (x 10^{12}/L):

Hemoglobin (g/dL):

Platelets (x 10^9/L):

Leukocytes (%):

Neutrophiles (x 10^9/L):

Eosinophiles (x 10^9/L):

Basophiles (x 10^9/L):

Lymphocytes (x 10^9/L):

Monocytes (x 10^9/L):

Biochemistry

Glucose (mg/dL):

Total Cholesterol (mg/dL):

Uric acid (mg/dL):

Creatinine(mg/dL):

HDL Colesterol (mg/dL):

Sodium (mmol/L):

Urea (mg/dL):

Triglycerides (mg/dL):

Potassium (mmol/L):

Chlorides(mmol/L):

AST/SGOT (U/L):

ALT/SGPT (U/L):

Free PSA (ng/L):

Total PSA (ng/L):

Glycated hemoglobin (Hb A1c - %):

Urine

pH reaction:

Glucose (mg/dL):

Other alterations:

Gleason score: Select one of the options

Prostatic neoplasia stage (according to TNM classification):

Select one of the options

Select one of the options

Select one of the options

If other, please specify:

Description of Prostate Ultrasound (if available):
